# New Drugs and Appliances

**Published:** 1894-12-08

**Authors:** 


### NEW DRUGS AND PREPARATIONS.

[We shall be glad to receive, at our Office, 428, Strand, London, W.C., from the manufacturers, specimens of all new preparations and drugs which may be brought out from time to time.]

### ICHTHYOL COLLODION.

Retrini has successfully treated two cases of acute rosacea in young subjects with the following mixture: Resorcin, gr. 15; ichthyol, gr. 30; collodion flex., oz. 1. The affected parts were painted with the liquid, which was allowed to dry and form a thin layer over them, any pustules present having been previously evacuated. The application was repeated three days in succession. Five or six days after the layer began to crack, and peeled off about two days later. From the first the local condition improved, and the redness and size of the nodules diminished. This was repeated two or three times, and recovery had taken place in about three weeks. (Nouv. Remèdes, Sep. 8th, 1893.)

